# Comparison of *Plasmodium berghei *challenge models for the evaluation of pre-erythrocytic malaria vaccines and their effect on perceived vaccine efficacy

**DOI:** 10.1186/1475-2875-9-145

**Published:** 2010-05-27

**Authors:** Wolfgang W Leitner, Elke S Bergmann-Leitner, Evelina Angov

**Affiliations:** 1 Malaria Vaccine Development, Walter Reed Army Institute of Research, Silver Spring, MD 20910, USA; 2 NIAID, NIH, Bethesda, MD 20892, USA

## Abstract

**Background:**

The immunological mechanisms responsible for protection against malaria infection vary among *Plasmodium *species, host species and the developmental stage of parasite, and are poorly understood. A challenge with live parasites is the most relevant approach to testing the efficacy of experimental malaria vaccines. Nevertheless, in the mouse models of *Plasmodium berghei *and *Plasmodium yoelii*, parasites are usually delivered by intravenous injection. This route is highly artificial and particularly in the *P. berghei *model produces inconsistent challenge results. The initial objective of this study was to compare an optimized intravenous (IV) delivery challenge model with an optimized single infectious mosquito bite challenge model. Finding shortcomings of both approaches, an alternative approach was explored, *i.e*., the subcutaneous challenge.

**Methods:**

Mice were infected with *P. berghei *sporozoites by intravenous (tail vein) injection, single mosquito bite, or subcutaneous injection of isolated parasites into the subcutaneous pouch at the base of the hind leg. Infection was determined in blood smears 7 and 14 days later. To determine the usefulness of challenge models for vaccine testing, mice were immunized with circumsporozoite-based DNA vaccines by gene gun.

**Results:**

Despite modifications that allowed infection with a much smaller than reported number of parasites, the IV challenge remained insufficiently reliable and reproducible. Variations in the virulence of the inoculum, if not properly monitored by the rigorous inclusion of sporozoite titration curves in each experiment, can lead to unacceptable variations in reported vaccine efficacies. In contrast, mice with different genetic backgrounds were consistently infected by a single mosquito bite, without overwhelming vaccine-induced protective immune responses. Because of the logistical challenges associated with the mosquito bite model, the subcutaneous challenge route was optimized. This approach, too, yields reliable challenge results, albeit requiring a relatively large inoculum.

**Conclusions:**

Although a single bite by *P. berghei *infected *Anopheles *mosquitoes was superior to the IV challenge route, it is laborious. However, any conclusive evaluation of a pre-erythrocytic malaria vaccine candidate should require challenge through the natural anatomic target site of the parasite, the skin. The subcutaneous injection of isolated parasites represents an attractive compromise. Similar to the mosquito bite model, it allows vaccine-induced antibodies to exert their effect and is, therefore not as prone to the artifacts of the IV challenge.

## Background

Despite decades of research, malaria infection remains a major global health problem with high mortality and morbidity. Efforts to disrupt the life cycle of the parasite by controlling the vector have had only limited success, while the usefulness of anti-malarial drugs is hampered by their lack of availability to those most in need and to the rapid evolution of drug resistant parasites. An effective vaccine remains the most promising approach to controlling the disease [1], but is still not available.

Research on malaria vaccines is complicated by the lack of surrogate markers of protection and the complexity of the parasite life cycle. To complicate matters, reported mechanisms of protection against the liver stage of the parasite differ not only between human and rodent malaria species, but also between different rodent malaria species. Therefore, infection of vaccinated animals with live parasites, as has previously been used to evaluate anti-malarial drugs, remains the most, if not only, meaningful readout of vaccine efficacy [2,3]. Such a challenge model should meet the following requirements: 1) reproducible infection of a predictable proportion of the challenge cohort; 2) establishment of infection using numbers of parasites consistent with natural infection, and 3) a predictable time to patency. The most frequently used method for challenging animals with malaria is the intravenous (tail vein) injection of parasites isolated from the salivary glands of *Anopheles *mosquitoes. This method was first described for the *Plasmodium berghei *model almost four decades ago [4] and has not significantly evolved since. In this method, mice are injected with a defined number of sporozoites. However, the number of sporozoites required to infect 90-100% of the mice ranges from 30 to 5,000 sporozoites. This number also varies widely from day to day, from mouse strain to mouse strain and from lab to lab (personal observation and [4-7]). The significant variability may be a consequence of changes in sporozoite virulence or to host response to extraneous materials in the inoculum such as mosquito debris, salivary gland flora, or dead sporozoites [8]. In addition, differences reported by different laboratories are presumably exacerbated by the use of laboratory-specific isolates and sub-strains of the parasite. Obtaining consistent IV infection of the frequently used BALB/c mice has been especially problematic. Scheller and colleagues approached establishing an effective challenge model by using gradient centrifugation to purify parasites [6]. Purification of sporozoites by this method produced more reproducible infections, but also required 5,000 sporozoites per inoculum, which is far larger an amount than the 25 to 250 sporozoites estimated to be inoculated during a mosquito bite challenge [9-11].

In addition to the technical problems associated with the IV challenge model, the approach also raises very serious scientific concerns. In contrast to the IV injected parasites, *Plasmodium *delivered by their arthropod vector, the *Anopheles *mosquito, are not injected directly into the blood stream, but rather into avascular portions of the skin from where they travel into the liver over an extended period of time [11,12]. It is unclear how this transient existence of the parasite in host tissue outside the hepatocytes affects its viability. Recent evidence suggests that a good proportion of injected sporozoites either remain close to the entry site in the dermis or reach the lymph node [11]. It remains to be determined what the consequence of these different pathways is for mounting of a host immune response. Convincing evidence already exists that bypassing this extra-hepatic phase can lead to unacceptable conclusions regarding effector mechanism when analyzing vaccine-induced protection against liver stage infection. For instance, in one study, sporozoites delivered to the skin by a mosquito were rapidly immobilized by sporozoite-antigen specific antibodies while still in the skin [13]. In another study where the IV challenge was used to determine the effector mechanism of a genetically-attenuated sporozoite vaccine, CD8^+ ^T-cells were implicated in immune protection [14]. This conclusion is likely incomplete since vaccine-induced antibodies did not have the opportunity to exert their effect as the transit time of the IV injected parasites from injection site to the liver was too short [15]. The impact of the challenge route on the efficacy of a passive (monoclonal anti-CSP antibodies) *P. berghei *vaccine was also observed in a side-by-side comparison of the IV and mosquito bite challenge in the *P. berghei *model of CD-1 mice [16]. The outcome of this study, however, was reversed with antibody-injected mice appearing better protected after IV challenge than mosquito bite infection. In the *Plasmodium yoelii *model, others were unable to obtain reliable challenge results in BALB/c mice by using varying numbers of infected *Anopheles stephensi *mosquitoes [17], which is unexpected in light of the sensitivity of this mouse strain to *P. yoelii *[7]. Despite the obvious shortcomings, the IV challenge model nevertheless continues to be used for reporting malaria vaccine efficacy [18-20].

An alternative challenge approach introduced several decades ago is through the bite of an infectious mosquito, the natural delivery route of transmission [21]. This is also the method of choice for the challenge of human volunteers in *P. falciparum *vaccine trials [22-25]. To date, no candidate vaccine has shown reproducible field efficacy without demonstration of efficacy in a clinical challenge trial using this approach [26]. However, the mosquito bite challenge is rarely employed in mouse models of malaria. The primary reasons for this are that it is more labour intensive requiring more and better trained personnel, and well established conditions for a mosquito bite challenge of mice are not readily available. While the number of sporozoites delivered by a single bite of an infected mosquito may still not precisely be known [9,10], another study using PCR to detect parasite RNA in the liver forty hours after a blood meal concluded that the inoculum from a single mosquito bite is quite consistent [27]. These observations indicated the need for optimizing the mosquito bite challenge model to consistently deliver the same inoculum. This can be accomplished by only challenging with a single infected mosquito. Protective immunity may be overwhelmed when using the previously described bulk-challenge method [28] in which a pool of mosquitoes with an unknown and variable infected portion are allowed to feed.

The objective of this study was to first optimize and then compare the IV with the single mosquito bite challenge method and characterize the parameters required to reliably challenge mice. The mosquito bite challenge method described here was successfully used to determine the efficacy of the first *P. berghei *CSP DNA vaccine [29,30]. The single mosquito bite challenge is clearly superior to the IV injection of isolated parasites thus confirming the conclusions of others who only used the CD-1 mouse model [16]. However, the increased need for manpower and longer duration of a mosquito bite challenge, especially for large-scale vaccine studies represent a significant hurdle for its acceptance. Therefore, attention was focused on a compromise approach, the subcutaneous injection of isolated parasites into the inguinal site of mice [31-33]. This approach combines the targeting of the correct anatomical site for the delivery of parasites with the ease of the intravenous injection, while eliminating the need to prepare the mice for the procedure (*i.e*., warming them for IV injection or anesthetizing them for mosquito-bite challenge) and produces highly reproducible challenge outcomes.

## Methods

### Mice and parasites

Female, 4-6 wk-old, BALB/c, C57BL/6, and ICR (CD-1) mice were obtained from The Jackson Labs. CD-1 mice for the subcutaneous challenge experiments were from Harlan Sprague-Dawley. ANKA strain *P. berghei *parasites were maintained at the Division of Entomology (WRAIR). For needle-based challenges, parasites were isolated from mosquito salivary glands 26 to 28 days after infection without further purification.

### IV challenge

Parasites were delivered by using a 1.0 ml tuberculin syringe and a 30 gauge needle. Sporozoites were dissected into M199 medium (GIBCO/Invitrogen) containing 5% normal BALB/c mouse serum (Harlan Bioproducts for Science Inc., Indianapolis, IN) which was standardized and pathogen screened. All further manipulations and dilutions of sporozoites were with this medium. Diluted sporozoites were maintained on ice until use within two hours. For challenge, mice were warmed via a heat lamp and injected IV with 100 μl of a sporozoite suspension containing from 10-10,000 sporozoites.

### Mosquito bite challenge

*Anopheles stephensi *mosquitoes were placed into small, sealed, screened containers. Mice were sedated with a 0.1 ml intraperitoneal injection of a mixture containing 25 mg/ml Ketaset^® ^(ketamine HCL, Wyeth/Pfizer, New York, NY) and 5 mg/ml Rompun^® ^(xylazine HCL, Bayer, Pittsburgh, PA) in sterile saline. Sedated animals were placed individually on the containers until it was observed that a blood meal had been taken. The sex and number of the mosquitoes in each carton were monitored and controlled during all experimental procedures. An infectious mosquito bite was confirmed by dissection of the mosquito salivary glands and viewed by phase-contrast microscopy. If no sporozoites were observed, this process was repeated until an infected mosquito was identified.

### Subcutaneous inguinal challenge

Parasites were isolated and injected as described above for IV challenge, but delivered to the subcutaneous pouch at the base of the hind leg of mice. This route was chosen because it is easily accessible and holds larger amounts of liquid than other subcutaneous sites.

### Monitoring infection

Blood stage infection was determined in challenged animals by Giemsa-stained thin blood films. Thin blood films were prepared on days 7 and 14 by removing a less than 1 mm section of the distal end of the tail and spotting approximately 3 μl of blood onto a microscope slide. Slides were air dried, fixed with 100% methanol and stained with a 10% Giemsa solution for 10 minutes at room temperature. Slides were evaluated at a 1000× magnification (oil-immersion) using a Nikon E400 Eclipse microscope by reading 20 fields per slide.

### Vaccination of mice with a DNA vaccine

In order to evaluate the challenge models under vaccine trial conditions, mice were vaccinated with DNA vaccines encoding the *P. berghei *CSP gene previously reported to protect mice against challenge [29,31]. Briefly, a plasmid containing the full length CSP-gene of *P. berghei *[29] (designated here CSP(+)) was used for the initial experiments. For subsequent independent experiments, a plasmid encoding the full length CSP without the GPI anchor sequence (designated CSP(-A)) was employed. The CSP(-A) plasmid yields increased vaccine efficacy compared to the full length CSP(+) construct [34]. Plasmids were grown in *E. coli *and purified by double banding on cesium chloride gradients followed by chromatography on Qiagen columns (Valencia, CA) according to manufacturer's recommendation. DNA was precipitated on gold beads and used to vaccinate mice epidermally by gene gun delivery targeting two sites of the shaved abdominal area with 1 μg DNA dose each. When using the CSP(+) plasmids, animals were immunized two times at four-week-intervals and challenged two weeks after the last immunization. When using the CSP(-A) plasmid, mice received three immunizations at four-week intervals.

### Statistical analysis

In order to achieve an 84% chance to detect a statistical difference between an infection rate of 80% (a negative control rate) and an infection rate of 10% (a non-control infection rate), using Fisher's exact test (2-sided, with alpha = 0.05), ten mice per group were required (nQuery Advisor 6.0 software, Statistical Solutions, Saugus, MA).

## Results

### Challenge efficiency for IV and mosquito bite challenge

The number of IV injected sporozoites required to reliably challenge all or most (*i.e*., at least 90%) naïve controls (referred to as "challenge controls" thereafter to distinguish from vaccination controls) varies widely in the literature. For all IV challenge studies performed, a range of sporozoite doses was used (100 - 10,000) that included those reported by others. These data reveal that a standard IV challenge dose of 300 sporozoites infected 90% or more of naive BALB/c mice in 11 of 18 experiments (63% of the time). For these challenges, the mean ID50 (defined as the calculated number of sporozoites required to infect 50% of naive mice) was 50 ± 15 sporozoites (Table 1). In the remaining experiments with BALB/c mice, less than 90% of the challenge controls were infected, with a mean ID50 of 259 ± 78 sporozoites. The mean ID50's for infecting naïve C57BL/6 and ICR (CD-1) mice was 19 and 18 sporozoites, respectively.

**Table 1:** Comparison of intravenous vs. single mosquito bite challenge models

	IV Challenge			Single Mosquito Bite ^*e)*^		
	***Average***	***Standard***	***Success***	***Average***	***Standard***	***Success***
***Mouse Strains Tested***	***LD(50) ***^***a)***^	***Error***	***Rate ***^***b)***^	***% Infected***	***Error***	***Rate***
BALB/c *(Success)*	50^*c)*^	15	63%	98 ^*d)*^	1.3	80%
BALB/c *(Failed)*	259 ^*c)*^	78	N/A	75 ^*d)*^	5	N/A
C57BL/6	19 ^*c)*^	0	100%	100 ^*d)*^	0	100%
ICR (CD-1)	18 ^*c)*^	0	100%	100 ^*d)*^	0	100%

a) ID50's were calculated by the Reed-Muench method [46]

b) Success was defined as 90% or better of the naïve animals (challenge controls) being infected

c) Data obtained from 18 (BALB/c) or two (C57BL/6 and ICR (CD-1)) independent IV challenge experiments

d) Data obtained from 11 (BALB/c) or two (C57BL/6 and ICR (CD-1)) independent mosquito bite challenge experiments

e) Mice were challenged by a single infected female mosquito in the presence of two male mosquitoes (as described in Table 2)

By using the single mosquito bite challenge in BALB/c mice, a mean of 98% of challenge controls were infected in at least 80% of the challenge experiments performed. In replicate experiments with C57BL/6 and ICR (CD-1) mice, 100% of naïve animals were infected 100% of the time (Table 1).

### Conditions for an effective single bite challenge

In initial experiments, variations in feeding times by the single female mosquito in the container were observed. Interestingly, these variations seemed to be affected by the presence of male mosquitoes, which were often unintentionally co-transferred to the feeding container. To address this observation, the time between mosquito transfer to the container and initiation of feeding (probing) as well as the overall feeding time of a single malaria-infected or non-infected female mosquitoes were determined in the presence of varying numbers of male or female mosquitoes (Table 2). Age and batch-matched infected female mosquitoes required at least 45% more time to consummate a blood meal than did non-infected females. Adding two male mosquitoes to the challenge carton reduced the mean feeding time for an infected female to rates that were equivalent to those of single non-infected females, but had no effect on feeding time of an uninfected female. Although the addition of two males failed to increase the rate of probing by the infected female during feeding, it did reduce the time until feeding was completed. In contrast, the addition of uninfected females to the challenge cartons did not affect the time that an infected female required to feed.

**Table 2:** Sex mixed feeding populations increase mean feeding times

	**Exp. 1**	**Exp. 2**
**Mosquito feeding population ^*b)*^**	**Average time^a)^** **± SE**	**Average time^a)^** **± SE**
	
1 Infected ^c) ^+ 2 Uninfected Females	11.5 ± 4.3	7.7 ± 1.3
1 Infected + 1 Uninfected Female	8.7 ± 2.9	ND
1 Infected Female + 2 Males	2.4 ± 0.8	5.4 ± 0.8
1 Infected Female + 1 Male	6.2 ± 2.9	ND
1 Uninfected Female	2.4 ± 4.6	5.5 ± 3.2
1 Infected Female	8.7 ± 1.8	8.8 ± 2.4

a) Average feeding time in minutes (*i.e*, time between start of actual blood meal and retraction of the proboscis, excluding time spent on probing)

b) n = 10 BALB/c mice for each group

c) Infection status of "infected" mosquitoes was confirmed after a successful blood meal

### Categorization of IV challenge failures

**Figure 1:**
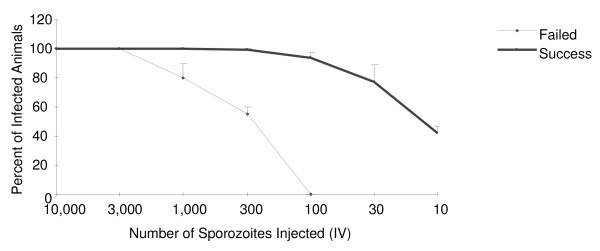
**Titration of sporozoites for intravenous challenge**. Naïve BALB/c mice (10 per group) were challenged with titered numbers of *P. berghei *SPZ by IV (tail vein) injection. Data are compiled from 18 independent challenge experiments. Challenges were arbitrarily considered to have been successful (solid line) when at least 90% of the mice challenged with 300 SPZ were infected (as determined by parasitemia 14 days after challenge) or failed (dotted line) when less than 90% of mice challenged with 300 SPZ were infected. N = 10 mice/group for each experiment. Shown are the average numbers of infected animals from independent challenge experiments ± SEM.

As expected, the mean titration curve associated with BALB/c mouse challenges in which the ID50 was 50 sporozoites (successful challenge) was different from experiments where the ID50 was 259 (failed challenge)(Table 1 and Figure 1). Although the curves indicate that a discrete variable may be associated with sporozoite virulence, it could not be identified in this study. Variables that were tested include mouse age, the nature of the protein support used during dissection and dilution (foetal bovine or normal mouse serum, heat inactivated foetal bovine or mouse serum, bovine serum albumin), pre-feeding infected mosquitoes with a blood meal prior to dissection, and the duration of time in which the parasite suspensions were held on ice before use. When comparing different sources of serum protein support, fresh mouse serum was found to promote maximal sporozoite viability (obtained from littermates of experimental animals) compared to commercially obtained or previously frozen mouse serum.

**Figure 2:**
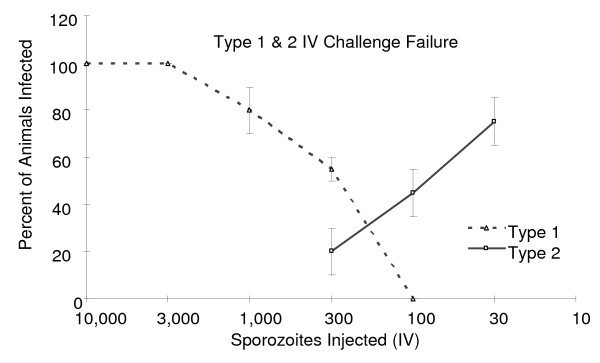
**Analysis of failed IV challenge experiments**. Failed (*i.e*., less than 90% of naïve mice challenged with 300 SPZ became infected) challenge experiments showed one of two titration patterns. Type 1 challenge failures (dotted line) showed a direct relationship between sporozoites dose and percent of mice infected. Type 2 failures (solid line) showed an inverse relationship between sporozoites dose and the percentage of mice infected. N = 10 mice/group for each experiment. Shown are the average numbers of infected animals from independent challenge experiments ± SEM.

When an IV challenge failed because less than 90% of challenge control animals were infected, two types of challenge failures were observed. The first type has been discussed above, where the ID50 for the challenge was 259 sporozoites (Type 1 challenge failure). For the second type of failure (Type 2 challenge failure), the ID50 could not be calculated because infectivity and challenge dose were inversely related (Figure 2) suggesting the presence of a parasite- or mosquito-derived contaminant. Type 2 challenge failures appeared to correlate with the use of parasite preparations that required a larger than average number of infected mosquitoes in order to obtain the necessary number of sporozoites. Such preparations are expected to contain higher concentrations of mosquito debris and/or non-infectious sporozoite material.

### Varying IV challenge strength and its impact on reported vaccine efficacy

The reproducibility of a challenge is a central prerequisite for any vaccine study. The requirement to obtain high quality sporozoites for use in a challenge can be associated with varying sporozoite viability, which then may affect the protective efficacy induced by a vaccine. When using the IV challenge model, even within experiments which were defined as 'successful challenges' (where 90% or more of challenge controls were infected), comparing the outcomes from these different challenge experiments was not possible (Table 3). In one experiment, mice were vaccinated two times with a DNA vaccine by epidermal injection and challenged with 300 sporozoites IV two weeks after the last immunization. This vaccination regimen was chosen to avoid maxing out the antibody-dependent protection induced by this vaccine [29]. The ID50 for this challenge was 178 sporozoites and 70% of the vaccinated mice were protected (p = 0.01), suggesting that this vaccine and the vaccine regimen used were effective. In an independent repeat experiment, three identical groups of vaccinated mice were challenged with 30, 100, or 300 sporozoites on the same day. The ID50 for this challenge was less than 30 sporozoites. The apparent increased infectivity of sporozoites used for the second experiment seemed to overwhelm the protective effects demonstrated in the first experiment, and thus reported poor vaccine efficacy. This, however, was contrary to the overwhelming evidence from other challenge experiments that established the efficacy of this particular vaccine.

**Table 3:** Inter-experimental variations of IV challenge and effect on perceived vaccine efficacy

Experiment 1	*% Infected, p *^*b)*^	*% Infected*	***SPZ***.	*Challenge*
	*CSP(+) *^*d)*^	*Naïve*	*LD(50) )*^*e*^	*Status*
	
Chall. w/300 SPZ ^c)^	30, p = 0.01	90	178	Success ^a)^
				

**Experiment 2**	***% Infected, p***	***% Infected***	***SPZ***.	***Challenge***
	***CSP(+)***	***Naïve***	***LD(50)***	***Status***
	
Chall. w/30 SPZ	70, p = 0.11	100	ID50 < 30	Success
Chall. w/100 SPZ	80, p = 0.24	100	ID50 < 30	Success
Chall. w/300 SPZ	80, p = 0.24	100	ID50 < 30	Success

a) Success was defined as being able to infect 90% or better of the challenge control animals.

b) p was calculated by Fisher-Yates Exact 2 × 2 test with native controls.

c) All experimental groups consisted of 10 BALB/c mice.

d) DNA plasmid that encodes a full-length *P. berghei *circumsporozoite protein.

e) ID50's were calculated by the Reed-Muench method [46] from naive titration controls.

### The subcutaneous challenge as an alternative to the intravenous and bite challenge model

The subcutaneous injection of isolated sporozoites has been proposed as an alternative to either of the two methods described above since it appears to combine the advantages of both. This method has already been used successfully to test the efficacy of various *P. berghei *DNA vaccine constructs [31-33]. When determining the minimal dose required for a reliable infection of at least 90% of naïve animals it became evident that a larger number of isolated sporozoites was required than needed for the IV challenge to infect BALB/c mice (Table 4). Interestingly, the titration curves obtained with the subcutaneous challenge of BALB/c and ICR (CD-1) mice never resulted in a plateau, but consistently produced a bell-shaped curve. A standard dose of 5,000 sporozoites was chosen for BALB/c mice. Challenge doses required to infect C57BL/6 and ICR (CD-1) mice by the subcutaneous route were 100 and 15,000, respectively (Table 4).

**Table 4:** Sporozoite titration for subcutaneous challenge

C57BL/6^a)^		BALB/c		ICR (CD-1)	
		
SPZ	% infected	SPZ	% infected	SPZ	% infected
30	60%	1,000	0%	1,000	0%
100	100%	2,000	0%	3,000	0%
300	100%	3,000	60%	5,000	0%
		4,000	90%	10,000	80%
		5,000	90%	15,000	100%
		8,000	80%	20,000	0%
		10,000	0%		

a) N = 5/group for C57BL/6 and ICR (CD-1), N=10/group for BALB/c. Shown is one representative titration experiment. Results were confirmed in independent repeat experiments.

### The influence of challenge conditions on perceived vaccine efficacy

In contrast to the subcutaneous challenge, which reliably infects mice when using a 'standard' dose of *P. berghei *sporozoites, the inoculum required to infect through the IV route can vary significantly. This raised the question what impact the size of the parasite inoculum might have on the perceived vaccine efficacy. For this purpose, the experiment described in Table 3 was repeated with the optimized CSP plasmid, pCSP(-A) (Table 5). This plasmid consistently protects the majority or all immunized BALB/c mice from infection by subcutaneously delivered sporozoites through an antibody-dependent mechanism without the involvement of effector T cells [30,31,33,34]. This route was used as a reference and positive control. Mice immunized with either the control plasmid (pcDNA) or the CSP-plasmid (CSP(-A)) were challenged by subcutaneous injection of a fixed dose or a titered number of sporozoites by the intravenous route. The subcutaneous challenge confirmed the high (100%) efficacy of the CSP-based DNA vaccination. However, the observed vaccine efficacy in IV challenged mice was dependent on the challenge dose. Despite the fact that the challenge was considered "successful" based on (a) the number of infected challenge controls at the 300 SPZ dose, and (b) the ID50 which was consistent with that previously established in BALB/c mice (Table 1), the perceived vaccine efficacy was only 44% (for 300 SPZ) and as low as 12.5% when challenging with 1,000 SPZ.

**Table 5:** Effect of IV challenge dose on perceived vaccine efficacy

	**pcDNA^a)^**	**pCSP(-A) ^a)^**	
		
**Challenge**	**Infected/total**	**Infected/total**	**Efficacy**
	
10,000 SPZ/IV	10/10	8/10	20%
3000 SPZ/IV	9/9	7/10	22%
1000 SPZ/IV	8/10	7/10	12.5%
300 SPZ/IV	9/10	5/10	44%
100 SPZ/IV	7/10	0/10	100%
			
5000 SPZ/SQ	9/10	0/10	100%

a) Three DNA immunizations of BALB/c mice delivered by gene gun (3 shots/immunization, 1 μg plasmid/shot), empty control plasmid (pcDNA) or CSP-encoding plasmid (plasmid and immunization described previously [30,34]. All animals were challenged with sporozoites from the same batch of infected mosquitoes. Results were confirmed in independent repeat experiments

To further investigate the effect of the challenge route on the perceived vaccine efficacy, BALB/c mice were immunized twice by either intramuscular injection (50 μg/dose) or gene gun bombardment of the skin (3 shots/immunization, 1 μg/shot) using an empty plasmid (pcDNA) or the *P. berghei *CSP(-A) DNA vaccine [34], which has previously been shown to induce strong antibody responses and protective immunity that appears to be independent of effector T cells [33]. Mice were challenged in parallel either by the subcutaneous or IV route. The subcutaneous route is comparable to the mosquito bite challenge in that the inoculum is delivered to the same tissue site (skin) where, in contrast to the IV challenge model, the sporozoites are capable of interacting with antibodies for extended periods of time [13]. As predicted by Vanderberg *et al *[15] the challenge route significantly affected the level of protection. CSP-plasmid immunized BALB/c mice were protected from 5000 subcutaneously delivered sporozoites, but poorly/not protected from a significantly smaller inoculum (300 SPZ) of intravenously injected sporozoites (Table 6).

**Table 6:** Effect of challenge route on perceived vaccine efficacy

	**Infected/total mice**	
**Groups**	**pCSP(-A)^a)^**	**pcDNA ^a)^**
	
gene gun vaccination, SQ challenge	0/10	9/10
gene gun vaccination, IV challenge	15/15	12/12
		
IM vaccination, SQ challenge	5/10	ND
IM vaccination, IV challenge	15/15	13/13

a) BALB/c mice were immunized by gene gun or intramuscular injection with plasmid encoding CSP or empty vector. 10-15 mice/group were challenged through the subcutaneous (with 5,000 SPZ) or intravenous (with 300 SPZ) route using the same batch of isolated *P. berghei *SPZ. Shown are the numbers of infected/total number of mice per group. The experiment was independently repeated using different, but overlapping challenge doses.

## Discussion

The immune mechanisms responsible for protecting against *Plasmodium*-infection or morbidity associated with chronic infection are surprisingly still not understood. This is in part due to insufficient efforts spent on the characterization of immune correlates of protection and to the fact that different mechanisms appear to be required to protect against different species of *Plasmodium*.

While cytotoxic T cells have been described as the main effectors protecting mice against *P. yoelii *infection after immunization with a CSP-based DNA vaccine [35], CTL appeared to be irrelevant in mice protected from *P. berghei *infection following immunization with a similar vaccine [33]. In the latter study, Th2-type antibodies recognizing specific epitopes on the CS-protein correlated with protection. Because of the lack of robust surrogate markers of protection, challenging with live parasites remains the most meaningful method to determine the potential protective efficacy of a candidate vaccine for both preclinical and clinical trials.

While vaccinated human volunteers are generally challenged through the bite of infected mosquitoes, the IV challenge model remains the most commonly used approach for infecting rodents with malaria because of the relative convenience and the ability to quantitate the sporozoite inoculum delivered. However, the number of sporozoites required to reproducibly infect 90-100% varies greatly. BALB/c mice, while frequently used for malaria vaccine studies, are particularly difficult to infect reliably. This may be due to the much higher susceptibility of this strain's innate immune system to *P. berghei *(but not to *P. yoelii*), which has been known for several decades [7] and had been used to question the usefulness of this mouse strain for *P. berghei *studies [4]. Malaria infection in general, and specific malaria-associated molecules, have been shown to trigger inflammatory responses through the stimulation of innate immune receptors [36-39]. Many such studies were, however, conducted *in vitro*, and while it may be questionable whether noticeable inflammatory responses are induced by these relatively small mosquito-delivered inocula, such responses can be expected to be amplified when a relatively large amount of parasite-derived and mosquito-derived debris are delivered together. Not surprisingly, such variability can also be attributed to seasonal factors that are generally not considered such as changing microbial flora delivered with the isolated sporozoites. Malaria parasites have repeatedly been found to be surprisingly susceptible to inflammation, either caused by another infection or by inflammatory mediators. The sensitivity of sporozoites to innate immune responses was shown by Nussler and colleagues 1993 who observed that irradiated *P. yoelii *sporozoites could produce a spontaneous intrahepatic inhibition of infection for *P. berghei *sporozoites that lasted up to three weeks after delivery of the last dose of irradiated *P. yoelii *and that this inhibition disappeared completely after one month [8]. Similarly, activators of innate immune responses such as CpG oligonucleotides, ligands for toll-like receptors and acute phase proteins have been shown to have a significant effect on host susceptibility and resistance against sporozoite infections [40-42].

When establishing the IV challenge model, it became evident that substantially higher numbers of sporozoites were required for complete infectivity in the three mouse strains that were tested than were observed by other investigators (Table 7). The need for fewer sporozoites to establish infection in the majority of naïve mice may be due to several modifications of previously published protocols. These differences to the procedures described by others include: (a) the use of mouse serum as the diluent; (b) keeping sporozoites on ice in cold M199 media containing 5% normal mouse serum (NMS) from the time of gland dissection until tail vein injection; and (c) the use of 30 gauge needles instead of 27 gauge needles for tail vein injection of sporozoite suspensions. Furthermore, sporozoites used for the studies presented here were not gradient-purified, which had been shown to produce more consistent inocula, but required significantly larger numbers of sporozoites likely due to the reduced viability of the purified sporozoites [6]. After thirty-seven independent experiments, the most reproducible challenge results were performed with sporozoites that were suspended and diluted with 5% NMS in M199 medium. Sporozoites were held in glass containers on ice until the time of injection. Under these conditions, a mean ID50 of 50 was reproducibly obtained.

**Table 7:** Examples of reported ID50 doses for infecting mice with *P. berghei *by IV challenge

**Mouse strain →**	**A/J**	**CD1/ICR**	**C57BL/6**	**BALB/c**
	
ID50 (ref [5])		3162	562	562
ID50 (ref [6])	2818	17,378	<30	3801

The single infectious bite challenge model was developed as an alternative to the relatively unreliable and unpredictable IV challenge method. This method does not require the inclusion of a large number of challenge control animals in each challenge experiment that need to be injected with various numbers of SPZ to be able to reliably determine the success and virulence of the challenge. This model proved to be significantly more reliable, requiring only a single *P. berghei *ANKA-infected mosquito to infect most or all challenge controls regardless of their genetic background. In contrast, another study reports the need for at least five mosquitoes to reliably infect 100% of CD-1 mice [16]. Although the reason for the discrepancy is unknown, the authors of this study nevertheless conclude that the mosquito bite challenge should be used rather than IV injection of parasites to avoid artifacts. For the *P. yoelii *model of malaria, a recent report suggests that the IV challenge is more reliable than the infection of BALB/c mice through mosquito bite, which in the hands of these investigators required multiple infected mosquitoes [17]. Based on theses unexpected results, the authors recommend the IV injection of sporozoites as the preferred infection method, however, ignoring the impact on perceived vaccine efficacies.

The method is, however, more labour intensive and this underscored the need for a reliable, but more efficient challenge procedure. The use of mixed-sex mosquito-feeding populations represented a significant improvement since it decreased the time required for an infected female mosquito to take a blood meal. Specifically, the addition of two male mosquitoes enhanced the feeding behaviour of the *P. berghei *infected female mosquito. This simple modification decreased the time required for a normal sixty- to seventy-mouse experiment to four to six hours for challenge. The observations are consistent with previous reports that male and female mosquito interactions can dramatically alter feeding behaviour [43,44]. Therefore, a sex-linked feeding motivator (*e.g*. pheromone, wing beat frequency, etc) that allows infected female mosquitoes to complete their blood meal more rapidly must be present when males and females are together, likely because feeding behaviour among female mosquitoes is directly linked to egg laying [45].

The present study sought to determine whether a modified mosquito-bite challenge approach in which mice are exposed to a single infected mosquito could be developed as a more reliable, reproducible and more biologically relevant alternative to the routinely used IV challenge model. After establishing standard parameters required for making this model highly reliable, it became evident that despite its effectiveness the logistical hurdles involved reduce its usefulness. It is significantly more time and labour consuming than an injection-based challenge, which limits the size of challenge experiments to around one hundred mice per experiment. Therefore, a method was explored that combines the advantages of the IV and mosquito bite models, the subcutaneous injection of sporozoites, which generates highly reliable challenge results. This route, though physiologically much more relevant than the IV route, surprisingly required larger numbers of sporozoites than did the IV route, and the size of the inoculum that is required to produce one hundred percent infectivity is within a relatively narrow range. Delivering an increasing inoculum through the subcutaneous route resulted in a bell-shaped challenge curve and the drop in challenge efficacy at doses higher than 4,000-5,000 sporozoites/mouse and is likely caused by the same factors responsible for the Type 2 failure of IV challenges, namely inflammatory responses triggered by excessive amounts of stimulators of innate immune responses within the inoculum.

## Conclusion

The intravenous injection of sporozoites generates highly variable and poorly reproducible challenge results. Confirming the concerns of others [15] this study also reveals that it drastically affects the perceived efficacy of a pre-erythrocytic vaccine. The single infectious-bite challenge as described here for *P. berghei *is the most reliable and most relevant readout method for testing the efficacy of malaria vaccines in animal models. Since it is time and labour intensive, the subcutaneous injection of isolated sporozoites at the base of the hind leg presents itself as an acceptable alternative. This method is rapid, produces highly consistent challenge results and is much more comparable to the natural route of malaria infection than the frequently used IV challenge route.

Both injection-based challenge methods require a careful titration of the inoculum in naive control mice. Such a titration is required for each IV challenge experiment to be able to judge the virulence of the inoculum and to avoid the misinterpretation of vaccination studies. However, the subcutaneous challenge route appears significantly less sensitive to the day-to-day variations in the inoculum thus allowing the use of an initially established "standard" dose.

## Competing interests

The authors declare that they have no competing interests.

## Authors' contributions

WWL planned and conducted the IV and bite challenge experiments, prepared the manuscript. ESB-L planned and conducted the subcutaneous challenge experiments, assisted in the preparation of the manuscript. EA assisted with the challenge experiments and the preparation of the manuscript. All authors read and approved the final manuscript.
